# Patient self-management of oral anticoagulation with vitamin K antagonists in everyday practice: clinical outcomes in a single centre cohort after long-term follow-up

**DOI:** 10.1186/s12872-020-01448-7

**Published:** 2020-04-10

**Authors:** M. Corrochano, B. Jiménez, J. Millón, I. Gich, M. Rambla, E. Gil, P. Caparrós, R. Macho, J. C. Souto

**Affiliations:** 1 Haemostasis and Thrombosis Unit, Hospital de la Santa Creu i Sant Pau, Barcelona, Spain; 2 Institut de Recerca. Hospital de la Santa Creu i Sant Pau (IIB-Sant Pau), Barcelona, Spain; 3 Clinical Epidemiology Department, Hospital de la Santa Creu i Sant Pau, Barcelona, Spain

**Keywords:** Management, Anticoagulation, Vitamin K antagonists, Clinical practice, Complications

## Abstract

**Background:**

Patient self-management (PSM) of vitamin K antagonists (VKA) seems a very promising model of care for oral anticoagulation in terms of efficacy and safety. In comparison with other management models of VKA therapy, the number of scientific publications supporting the advantages of PSM is more limited. Currently, most of the scarce information comes from randomized clinical trials. Moreover, a small number of studies have assessed PSM of VKA therapy in real life conditions.

**Methods:**

We analyzed clinical outcomes of 927 patients in a single center (6018.6 patient-years of follow-up). Recruitment took place between 2002 and 2017. All patients followed a structured training program, conducted by specialized nurses.

**Results:**

Fifty percent of individuals had a mechanical heart valve (MHV), 23% suffered from recurrent venous thromboembolism (VTE) or high-risk thrombophilia, and 13% received VKA therapy because of atrial fibrillation (AF). Median follow-up was 6.5 years (range 0.1–15.97 years), median age was 58.1 years (IQR 48–65.9) and 46.5% were women. The incidence of major complications (either hemorrhagic or thromboembolic) was 1.87% patient-years (pt-ys) with a 95% CI of 1.54–2.27. The incidence of major thromboembolic events was 0.86% pt-ys (95% CI 0.64–1.13) and that of major hemorrhagic events was 1.01% pt-ys (95% CI 0.77–1.31). The incidence of intracranial bleeding was 0.22% pt-ys (95% CI 0.12–0.38). In terms of clinical indication for VKA therapy, the incidence of total major complications was 2.4% pt-ys, 2.0% pt-ys, 0.9% pt-ys and 1.34% pt-ys for MHV, AF, VTE and other (including valvulopathies and myocardiopathies), respectively. Clinical outcomes were worse in patients with multiple comorbidities, previous major complications during conventional VKA therapy, and in older individuals. The percentage of time in therapeutic range (TTR) was available in 861 (93%) patients. Overall, the mean (SD) of TTR was 63.6 ± 13.4%, being higher in men (66.2 ± 13.1%) than women (60.6 ± 13.2%), *p* < 0.05.

**Conclusions:**

In terms of clinically relevant outcomes (incidence of major complications and mortality), PSM in real life setting seems to be a very good alternative in properly trained patients.

## Background

Oral anticoagulant therapy (OAT) with vitamin K antagonists (VKA) has been used for more than 60 years to prevent frequent and serious thromboembolic complications associated with pathological conditions such as cardiac valve replacement, atrial fibrillation, and venous thromboembolic disease. Inevitably, OAT causes an increased risk of hemorrhagic events in these patients [1]. It is necessary to maintain the anticoagulant effect within margins that are both effective and safe, balancing bleeding and thrombotic risk in order to minimize complications.

The measurement of the anticoagulant effect of the VKA is carried out by the prothrombin time (Quick time) and is expressed by international consensus of the Scientific Societies as International Normalized Ratio (INR). The optimal intervals of INR have been defined according to the pathology and indication for OAT. The incidence of serious complications is lower in patients who are within the recommended intervals (INR 2.0–3.5 for the majority of cases). The Time in Therapeutic Range (TTR) is a parameter that reflects the percentage of time in which the patient’s INR values were within a desired range [2].

Conventionally, INR controls for OAT management are carried out in hospitals and primary care centers, and dosage adjustment is performed by a physician. Nevertheless, other strategies for control such as patient self-testing and patient self-management have shown to be equally reliable and safe [3]. In patient self-testing (PST), patients perform INR testing by themselves with a point-of-care (POC) instrument and report results to a physician who will adjust the dose. PST has proven to be comparable to standard management in terms of preventing major clinical outcomes, and to improve quality of life of patients [4–6].

Likewise, in patient self-management (PSM), patients perform weekly INR controls by using POC devices but, also, after a training course, they must be able to adjust the doses of the drug to stay within their therapeutic range [7]. Few population-based studies have collected data in real life setting of this model of care [8–10]. Results showed that the incidence of complications is lower with PSM than standard care of VKA treatment. Regarding TTR, results seemed to be at least comparable. In a recent study comparing PSM and direct oral anticoagulants (DOACs), PSM has shown significantly less incidence of thromboembolic complications [11]. None of these studies contain data from Spanish patients.

In the Hospital de la Santa Creu i Sant Pau we have the largest cohort of Spanish patients in self-management of oral anticoagulation with VKA in a single center. This cohort was initiated in 2002, continuing a clinical trial called ACOA [12] that demonstrated the capacity of most patients to perform self-management of VKA, the great clinical applicability of this model, and the significant reduction of serious complications compared to conventional control. These results were corroborated in meta-analyses of randomized, controlled studies [3, 13]. The present paper aimed at describing the clinical outcomes of this single center cohort, after a long-term follow-up of 16 years.

## Methods

### Objectives

The main objective was to determine, in patients under self-management of VKA drugs, the incidence of:

- Major thromboembolic events (TE): deep vein thrombosis, pulmonary embolism, ischemic stroke or other arterial thromboembolic events.
- Minor but relevant TE (as considered in our study): myocardial infarction, transient ischemic attack, thrombophlebitis or other thromboembolic events.
- Incidence of major hemorrhagic complications: grades 3 to 5 according to the BARC scale [14].
- Incidence of clinically relevant, but not major, hemorrhagic complications (grade 2 of the BARC scale).
- Mortality, related or not, to anticoagulant treatment.

### Study population and PSM training

This was a retrospective and unicentric observational study (NCT 03532724). We analyzed patients under the self-management regime, who were trained and followed-up in the Haemostasis and Thrombosis Unit of the Santa Creu i Sant Pau Hospital between July 2002 and December 2017. In our settings, conventional management of OAT consists of the monthly determination of INR with a POC device at a specialized anticoagulation clinic. Dose adjustment is performed by a haematologist.

Our cohort of patients under self-management started with 194 patients from the ACOA trial [12] who remained in PSM after the end of the study. Recruitment of patients was not systematic. If patients met the criteria for enrollment, the decision to include them in the programme was made by the physician. Physicians prioritized patients who had had serious complications under the conventional control and who could improve their quality of life with this management strategy. Criteria for enrollment were: patients aged 18 years or older who had been receiving long-term anticoagulant therapy (for at least 3 months) under conventional control method. Patients with severe physical or mental illness and without a responsible caregiver were not be included, neither patients who could not understand the Spanish language.

All patients were required to attend an educational program and pass a final examination. This training course was similar to that published in 2005 for the ACOA clinical trial [12]. In brief, the program consisted in two teaching lessons of 2 h each, including basic skills on the use of a coagulometer, interpretation of INR values and dosing of VKA drug. Patients had to demonstrate that they could follow the PSM procedures in an adequate form. In addition, they were requested to demonstrate their skills in a final examination. The lessons were conducted by a specialized nurse. Disabled patients were accepted to perform PSM if a caregiver participated in the educational program and the examination. All patients used the portable coagulometer CoaguChek S or CoaguChek XS (Roche Diagnostics, Switzerland) equipped with Coaguchek PT-test strips. The requested frequency of INR home testing was once a week. Patients noted every INR result and corresponding VKA dose in a diary. Since starting the PSM programme, each patient or caregiver was visited in scheduled appointments at least once a year by our team of specialized nurses.

### Data acquisition

We performed a comprehensive retrospective search of clinical information from the following sources:

- Medical records from our hospital. We reviewed the follow-up of the Haemostasis and Thrombosis Unit professionals (doctors and nurses) as well as other specialities, visits to emergency, and hospitalizations.
- Shared clinical records from Catalonia, which collects information regarding assistance of public hospitals of Catalonia. This allowed to access patients’ medical reports since its initiation in 2008.
- Information collected in the computerized program of public primary care centers in Catalonia, which started in 1998 and was generalized in 2005.

The following data were obtained: age at start of PSM, gender, anticoagulant treatment indication, date of treatment initiation, drug used, complications prior to the onset of self-management, complications during conventional VKA therapy, INR therapeutic range, concomitant use of antiplatelet drugs, existence of comorbidities (high blood pressure, diabetes, gastro duodenal ulcer, cancer, chronic liver disease, ischemic heart disease, heart failure, peripheral vascular disease, chronic renal insufficiency, lipid disease, atrial fibrillation, chronic obstructive pulmonary disease and others), follow-up period and cause of self-management termination (death, non-adherence, cognitive impairment, voluntary withdrawal, change of treatment, end of anticoagulation indication, displacement, unknown, others). In addition, patients who were currently on PSM completed a survey on clinical events related to the anticoagulation treatment.

Complications occurring during periods of heparin treatment were not considered as related to the anticoagulant treatment (for example: bridging therapies or hospital admissions where the anticoagulant regimen was changed).

### Statistical analysis

Baseline characteristics of patients are presented as absolute and relative frequencies [N (%)] for categorical variables; and mean and standard deviation (mean ± SD) for quantitative variables. Person-time was calculated as the elapsed time between the initiated PSM and the event of interest or first coming of December 31, 2017: emigration, self-management termination by any reason or death. Clinical endpoints are expressed by incidence rates and the 95% confidence interval (95% CI), calculated as number of events divided by person-time at risk. The time in therapeutical range (TTR) was calculated by the Rosendaal method of lineal interpolation [15]. The individual TTR was crudely estimated i.e. without accounting for anticoagulation interruption during invasive procedures or bridging therapies. A non-parametric test (Mann-Whitney) was used for comparing the TTR. The 95% CI of the incidence were also calculated. Analyses were performed by using the Stata (V 15.1) statistics software package.

## Results

**Fig. 1:**
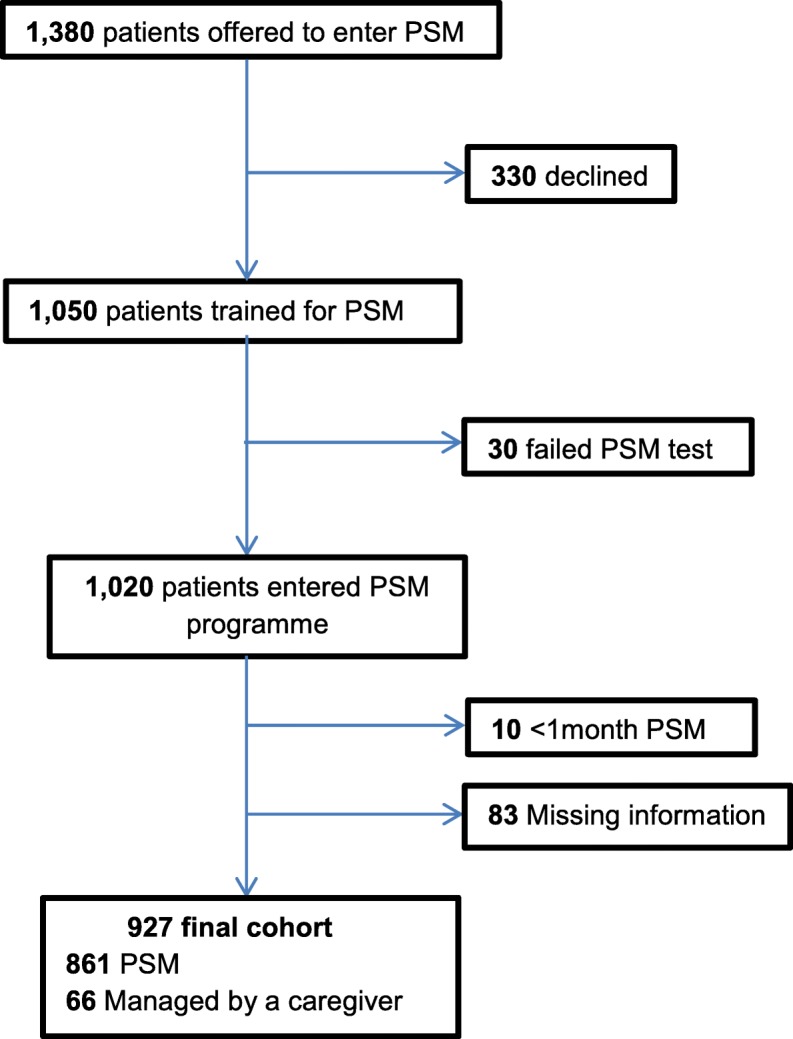
Study flow chart

A total of 1380 patients were initially offered to participate in the PSM programme, however, 330 (24%) declined. The remaining 1050 patients were trained for PSM, but 30 of them did not pass the final examination. Therefore, 1020 patients were included in the programme. Patients over 18 years of age were only included for analysis. We excluded patients with insufficient clinical information to evaluate the previously indicated variables, and those who remained less than 1 month under self-management (Fig. 1). The final cohort for analysis included 927 patients. Baseline characteristics of the overall population are shown in Table 1.

**Table 1:** Baseline characteristics of the population included in the study

		Indication for OAT					
	Overall	Atrial fibrillation	MHV	VTE	Cardiopathy	Valvulopathies	Other
**Patients** N (%)	927 (100)	120 (12.9)	465 (50.2)	213 (23.0)	36 (3.9)	41 (4.4)	52 (5.6)
**Age** (median - IQR)	58.1 [48–65.9]	66.3 [58.7–70.9]	60.8 [52.7–67.1]	47.6 [38.4–56.6]	52.7 [48.2–57.6]	62.6 [57.5–68]	48 [39.3–58.7]
**Female** (%)	431 (46.5)	29 (24.2)	234 (50.3)	108 (50.7)	8 (22.2)	30 (73.2)	22 (42.3)
**Time in traditional control of AVK, before PSM** (years ± SD)	8.5 ± 7.9	4.0 ± 4.1	11.4 ± 8.7	5.9 ± 6.1	6.4 ± 5.2	7.5 ± 6.1	5.0 ± 5.5
**Previous major complications N (%)**							
**Thromboembolic**	113 (12.2)	1 (0.8)	86 (18.5)	20 (9.4)	0 (0)	3 (7.3)	3 (5.8)
**Hemorrhagic**	62 (6.7)	5 (4.2)	46 (9.9)	4 (1.9)	0 (0)	1 (2.4)	6 (11.5)
**Any**	163 (17.6)	6 (5.0)	121 (26.0)	24 (11.3)	0 (0)	4 (9.8)	8 (15.4)
**Comorbidities N (%)**							
**Any**	754 (81.3)	110 (91.7)	418 (89.9)	115 (54.0)	33 (91.7)	39 (95.1)	39 (75)
**> 3**	202 (21.8)	32 (26.7)	129 (27.7)	10 (4.7)	12 (33.3)	11 (26.8)	8 (15.4)
**High blood pressure**	425 (45.9)	81 (67.8)	250 (53.8)	48 (22.7)	17 (47.1)	17 (41.5)	16 (31.7)
**Diabetes**	125 (13.4)	23 (19.1)	76 (16.3)	10 (4.6)	4 (10.5)	10 (24.4)	5 (10.0)
**Gastro duodenal ulcer**	50 (5.4)	10 (8.0)	31 (6.7)	5 (2.5)	2 (5.2)	2 (4.9)	1 (1.7)
**Cancer**	116 (12.5)	24 (19.9)	66 (14.2)	11 (5.3)	4 (10.5)	4 (9.8)	9 (16.7)
**Chronic liver disease**	30 (3.2)	2 (1.6)	23 (5.0)	2 (0.7)	1 (2.6)	1 (2.4)	2 (3.3)
**Ischemic heart disease**	93 (10.1)	22 (18.3)	50 (10.8)	5 (2.5)	8 (23.6)	2 (4.9)	7 (13.3)
**Heart failure**	126 (13.5)	18 (15.1)	86 (18.6)	5 (2.5)	12 (34.0)	5 (12.2)	3 (5.0)
**Peripheral vascular disease**	36 (3.9)	11 (8.8)	20 (4.4)	1 (0.4)	2 (5.2)	0 (0.0)	3 (6.7)
**Chronic renal insufficiency**	114 (12.3)	18 (15.1)	76 (16.3)	6 (2.8)	4 (10.5)	8 (19.5)	6 (11.7)
**Lipid disease**	405 (43.7)	65 (54.2)	237 (50.9)	56 (26.3)	14 (39.3)	22 (53.7)	14 (26.7)
**Atrial fibrillation**	279 (30.1)	8 (6.4)	222 (47.8)	7 (3.2)	18 (49.8)	29 (70.7)	7 (13.3)
**Chronic obstructive pulmonary disease**	74 (8.0)	21 (17.5)	35 (7.5)	6 (2.8)	6 (15.7)	3 (7.3)	4 (8.3)
**Others**	413 (44.6)	56 (47.0)	209 (45.1)	86 (40.5)	16 (44.5)	18 (43.9)	21 (40.0)

The total follow-up was of 6018.6 patient-years (pt-ys), mean (SD) follow-up was 6.5 ± 4.6 years (range 0.1–15.97 years). A total of 431 patients were female (46.5%), the median age (IQR) at the start of PSM was 58.1 years (48–65.9). There were 66 patients (7%) managed by a relative or third person (caregiver). As described before, in our center, patients trained for PSM were previously under conventional control. The mean time on conventional control before starting PSM was 8.5 ± 7.9 years.

The main indication for anticoagulation was prosthetic mechanical heart valve (MHV) in 465 patients (50.2%): aortic (*n* = 189,40.7%), mitral (*n* = 138,29.7%), pulmonary (*n* = 8,1.7%), tricuspid (*n* = 8,1.7%) and double prosthesis (*n* = 122, 26.2%). The following most frequent indications were: recurrent venous thromboembolism (VTE) (213 patients, 23%) and atrial fibrillation (AF) (120 patients, 12.9%).

Many patients included for analysis suffered from multi-pathological conditions: 754 (81.3%) had at least one comorbidity, and 202 (21.8%) had more than 3. Before starting PSM, 163 patients (17.6%) had experienced major complications on conventional control; 113 (12.2%) thromboembolic complications and 62 (6.7%) hemorrhagic ones. Twelve patients had suffered both types of major previous complications. (Table 1).

The most used drug was acenocumarol in 833 patients (89.9%); in the remaining, warfarin was used (94 patients, 10.1%). A total of 109 (11.8%) patients received, at any time during PSM, concomitant antiplatelet therapy, mainly aspirin (93 patients, 10%) and P2Y12 inhibitors (15 patients; 1.6%) and one patient received both.

Since the year 2010 we have had reliable data on TTR from 861 (93%) of patients. The overall mean (SD) TTR was 63.6 ± 13.4%. The TTR in women was lower than that of men: 60.6 ± 13.2% vs 66.2 ± 13.1% respectively (*p* < 0.05). TTR was correlated negatively with the target INR. It was significantly worse (*p* < 0.0001) as the INR target increased: INR 2.0–3.0 in 563 patients, TTR = 68.1 ± 11.3%; INR 2.5–3.5 in 251 patients, TTR = 58.1 ± 10.8%, and INR 3.0–4.0 in 13 patients, 45.5 ± 13.0%. In addition, TTR was significantly worse in patients with any major complication (56.0 ± 15.5%) than patients without them (64.5 ± 12.9), *p* < 0.0001.

### Complications during follow-up

A total of 106 (11.4%) patients had at least one serious complication during PSM. Three of them suffered both, one major hemorrhagic and one thrombotic. The overall incidence of serious complications (thrombotic and hemorrhagic) was 1.87% pt-ys (95% CI 1.54–2.27). The incidence of severe hemorrhagic complications was 1.01% pt-ys (95% CI 0.77–1.31) (*n* = 59), with an incidence of intracranial hemorrhage of 0.22% pt-ys (95% CI 0.12–0.38) (*n* = 13). The incidence of severe thrombotic complications was 0.86% pt-ys (95% CI 0.64–1.13) (*n* = 50). Incidence of thromboembolic complications was slightly higher in MHV, while incidence of major hemorrhagic complications was higher in atrial fibrillation patients (Table 2).

**Table 2:** Severe complications by indication and other relevant variables

	Any major event^**a**^	Thromboembolic	Major hemorrhagic	Intracranial bleeding	Transient ischemic attack	Mortality
	n; numbers per 100 patient-years (95% CI)					
**Overall (*n*** **= 927)**	106; 1.87 (1.54–2.27)	50; 0.86 (0.64–1.13)	59; 1.01 (0.77–1.31)	13; 0.2 (0.12–0.38)	17; 0.29 (0.17–0.46)	88; 1.46 (1.17–1.80)
**Indication**						
**Atrial fibrillation (*n*** **= 120)**	14; 2.00 (1.10–3.37)	3; 0.42 (0.09–1.22)	11; 1.57 (0.78–2.81)	6; 0.85 (0.31–1.86)	0	17; 2.34 (1.36–3.75)
**MHV (*n*** **= 465)**	71; 2.39 (1.87–3.01)	33; 1.06 (0.73–1.50)	41; 1.32 (0.95–1.79)	5; 0.16 (0.05–0.38)	14; 0.44 (0.24–0.74)	58; 1.79 (1.36–2.32)
**VTE (*n*** **= 213)**	11; 0.89 (0.45–1.60)	8; 0.64 (0.28–1.26)	3; 0.24 (0.05–0.69)	0	2; 0.16 (0.02–0.56)	5; 0.38 (0.13–0.91)
**Other indication (*n*** **= 129)**	10; 1.34 (0.64–2.47)	6; 0.78 (0.29–1.71)	4; 0.53 (0.14–1.36)	2; 0.26 (0.03–0.96)	1; 0.13 (0–0.73)	8; 1.04 (0.45–2.05)
**Male (*n*** **= 496)**	53; 1.80 (1.35–2.36)	19; 0.62 (0.38–0.97)	35; 1.17 (0.81–1.62)	8; 0.27 (0.11–0.52)	9; 0.29 (0.13–0.55)	51; 1.64 (1.22–2.16)
**Female (*n*** **= 431)**	53; 1.95 (1.47–2.56)	31; 1.11 (0.75–1.58)	24; 0.85 (0.55–1.27)	5; 0.18 (0.06–0.41)	8; 0.28 (0.12–0.55)	37; 1.27 (0.89–1.75)
**TTR < 55% (*n*** **= 199)**^**b**^	40; 3.66 (2.61–4.98)	24; 2.09 (1.34–3.11)	18; 1.54 (0.91–2.43)	2; 0.17 (0.02–0.62)	8; 0.67 (0.29–1.32)	22; 1.78 (1.12–2.70)
**TTR 55–64% (*n*** **= 216)**	22; 1.55 (0.97–2.35)	10; 0.68 (0.33–1.25)	12; 0.82 (0.43–1.44)	1; 0.07 (0–0.38)	4; 0.27 (0.07–0.69)	23; 1.53 (0.47–2.24)
**TTR 65–75% (*n*** **= 277)**	22; 1.16 (0.73–1.76)	10; 0.52 (0.25–0.95)	12; 0.62 (0.32–1.09)	2; 0.10 (0.01–0.38)	2; 0.10 (0.01–0.37)	17; 0.86 (0.50–1.38)
**TTR > 75 (*n*** **= 169)**	8; 0.77 (0.33–1.51)	2; 0.19 (0.02–0.68)	6; 0.57 (0.21–1.24)	4; 0.38 (0.1–0.97)	0	7; 0.65 (0.26–1.34)
**With previous severe complication (*n*** **= 163)**	35; 3.70 (2.56–5.12)	17; 1.71 (0.99–2.73)	19; 1.86 (1.12–2.90)	3; 0.29 (0.06–0.86)	9; 0.88 (0.40–1.67)	17; 1.59 (0.93–2.55)
**Without previous complications (*n*** **= 764)**	71; 1.51 (1.18–1.91)	33; 0.68 (0.47–0.96)	40; 0.83 (0.60–1.13)	10; 0.21 (0.10–0.38)	8; 0.16 (0.07–0.32)	71; 1.43 (1.12–1.81)
**> 3 comorbidities (*n*** **= 202)**	46; 3.36 (2.46–4.48)	19; 1.31 (0.79–2.04)	29; 2.01 (1.35–2.84)	5; 0.35 (0.11–0.81)	5; 0.33 (0.11–0.78)	51; 3.34 (2.44–4.40)
**≤ 3 comorbidities (*n*** **= 725)**	60; 1.40 (1.07–1.81)	31; 0.71 (0.48–1.00)	30; 0.68 (0.46–0.98)	8; 0.18 (0.08–0.36)	12; 0.27 (0.14–0.47)	37; 0.82 (0.58–1.14)
**Age < 40 (*n*** **= 134)**	11; 1.25 (0.63–2.24)	6; 0.66 (0.24–1.43)	6; 0.67 (0.25–1.46)	0	0	0
**Age 40–59.9 (*n*** **= 388)**	34; 1.34 (0.93–1.88)	15; 0.58 (0.32–0.96)	20; 0.77 (0.47–1.20)	3; 0.12 (0.02–0.34)	6; 0.23 (0.08–0.50)	31; 1.18 (0.80–1.67)
**Age 60–75 (*n*** **= 359)**	50; 2.46 (1.83–3.25)	27; 1.29 (0.85–1.87)	24; 1.12 (0.72–1.67)	3; 0.14 (0.03–0.41)	9; 0.42 (0.19–0.79)	43; 1.95 (1.41–2.63)
**Age > 75 (*n*** **= 46)**	11; 5.27 (2.63–9.42)	2; 0.84 (0.10–3.02)	9; 4.27 (1.95–8.1)	7; 3.32 (1.33–6.84)	2; 0.84 (0.10–3.04)	14; 5.82 (3.18–9.75)
**Patients managed by a third person (*n*** **= 66)**	15; 3.44 (1.93–5.67)	6; 1.30 (0.48–2.82)	9; 1.89 (0.86–3.59)	6; 1.26 (0.46–2.74)	2; 0.40 (0.05–1.46)	19; 3.77 (2.27–5.84)
**Patients under strict PSM (*n*** **= 861)**	91; 1.75 (1.41–2.14)	44; 0.82 (0.60–1.10)	50; 0.93 (0.64–1.23)	7; 0.13 (0.05–0.27)	15; 0.27 (0.15–0.45)	69; 1.25 (0.97–1.58)

We have grouped as “other”: cardiopathies, valvulopathies and other indications, because of small number of patients in each category

*CI* confidence interval

^a^ Any major event includes thromboembolic and/or haemorrhagic. ^b^ TTR was calculated only in 861 patients

A total of 162 patients (17.5%) suffered relevant complications (non-major): hemorrhagic (130 patients, 14%) and thrombotic (46 patients, 5%), or both (14 patients). Three patients suffered relevant thrombosis twice.

Incidence of complications (major and some relevant, such as transitory ischemic attack, TIA) as well as mortality are shown in Table 2. The incidences are displayed by relevant co-variables, such as indication for anticoagulation, gender, TTR, previous severe complications under conventional VKA treatment, comorbidities, age and management of OAT by a caregiver.

Regarding to indication of anticoagulation, the highest incidence of total complications was 2.39% pt-ys in carriers of MHV (95% CI 1.87–3.01), and the lowest in patients with recurrent VTE (0.89% pt-ys; 95% CI 0.45–1.60). The incidence of major complications was similar in men and women (1.80% pt-ys and 1.95% pt-ys respectively). Patients with higher TTR had around 4 times less incidence of complications than patients with the lower TTR: TTR < 55% the incidence of major complications was 3.66% pt-ys (95% CI 2.61–4.98) whilst patients with TTR > 75% had an incidence of 0.77% pt-ys (95% CI 0.33–1.51). Patients who had previous severe complications during conventional control had a higher incidence of major complications during PSM in contrast to those patients with no previous history (3.70% pt-ys versus 1.51% pt-ys, respectively). Elderly patients (> 75 years old) had an incidence of major complications of 5.27% pt-ys, 4 times higher than younger patients (< 40 years old) with an incidence of 1.25% pt-ys.

### Type of thrombotic complications

As mentioned, there were 50 major thrombotic complications. Thirty (60%) were cerebrovascular accidents, followed by prosthetic heart valve thrombosis and atrial thrombosis (*n* = 11), deep vein thrombosis (*n* = 8) and peripheral arterial embolism (n = 1).

There were 49 relevant thrombotic complications, in 46 patients. A total of 17 (35%) were transient ischemic attacks (TIA), followed by thrombophlebitis (*n* = 11), retinal thrombosis (*n* = 8), acute myocardial infarction (*n* = 7) and others (*n* = 6).

### Type and location of hemorrhagic complications

There were 59 major hemorrhagic complications. In 11 cases, the complication was secondary to trauma. Thirty-six complications (61%) were of gastrointestinal origin, of which 4 occurred in patients who received antiplatelet drugs concomitantly. Thirteen patients (22%) had intracranial bleeding, being secondary to a traumatic event in 9 cases. The incidence of intracranial bleeding was 0.22% pt-ys (95% CI 0.12–0.38). Other complications were: retroperitoneal (*n* = 4), gynecological (*n* = 3), pulmonary (*n* = 2) and urinary tract (*n* = 1) bleeding events.

There were 130 relevant hemorrhagic complications, 47 (36%) were secondary to a traumatic event, mainly epistaxis (*n* = 34; 26%), followed by intramuscular bleeding (*n* = 23; 18%), gastrointestional 15 (11%), Urinary tract 14 (11%), intra-articular 6 (5%), pulmonary 5 (4%), and other 33 (25%).

### Mortality

Overall incidence of mortality was 1.46% pt-ys (95% CI 1.17–1.80) (*n* = 88). Only 13 deaths were clearly related to VKA treatment; in 7 cases the cause of death was undetermined.

## Discussion

There is scarce data on PSM in real world settings. In previous similar papers, Nagler et al [8], presented a cohort of 1110 patients in Switzerland, with a median age of 54 years, followed-up a median of 4.3 years; and Nilsson et al [9]*,* published outcomes from 2068 patients in Denmark, with a median age of 49 years in women and 55 in men, followed-up a total of 6900 pt-ys. Incidences of major complications in both studies were similar to obtained in our cohort. The incidence of major hemorrhagic events was 1.1, 1.6 and 1.0% pt-ys, and major thrombotic events was 0.4, 0.7, 0.9% pt-ys (Nagler, Nilsson and our study, respectively; Nilsson did not include VTE events). The same happened with the incidence of intracranial bleeding: 0.2, 0.1, 0.2% pt-ys. Mortalities reported in the cohorts, related or not to OAT were 1.4, 0.5 and 1.5% pt-ys, respectively. These incidences (under PSM clinical model) are lower than reported from conventional management. Even the centers of recognized optimal managing of OAT such as the Swedish clinical network AuriculA report higher incidence of complications. Björck et al [16], published results from around 77,000 patients, corresponding to 217,000 pt-ys, managed in anticoagulation clinics (mean age 70) or primary health care centers (mean age 73), associated with a high standard in quality control (as demonstrated by TTR over 75%), and with very good clinical results in both settings: major bleeding incidence of 2.2% pt-ys, and thromboembolic events (excluding myocardial infarction) of 1.7% pt-ys. Of course, any comparison between different cohorts and different studies is indirect, and must be done cautiously, especially when indication for anticoagulation, ages or comorbidities are different.

We observed a slight increase in the incidence of complications when patients on PSM had lower TTRs, when they had more than three co-morbidities, when they had suffered previous severe complications under conventional VKA therapy, or when patients were managed by a caregiver. In this last circumstance, strictly speaking, we cannot consider the model as “patient self-management”. Other variable probably associated to worse outcomes is aging, since patients older than 75 exhibited an incidence of total complications 4 times higher than youngest (< 40 years old), mainly due to hemorrhagic events (incidences of 4.27% pt-ys vs 0.67% pt-ys). However, any comparison must be done very cautiously, since our sample size of older than 75 is very small (46 patients). It is worthy to highlight the very low incidence of intracranial bleeding of 0.22% pt-ys in our cohort, in comparison to reported with different antithrombotic therapies. Recently, Gulati et al [17], reported the risk of intracranial hemorrhages in users of antithrombotic drugs in Norway nationwide. Users of antithrombotic drugs (as a whole) had an incidence of 0.30% pt-ys, whereas for the different oral anticoagulants, the incidences were: warfarin 0.55% pt-ys, rivaroxaban 0.51% pt-ys, dabigatran 0.25% pt-ys, and apixaban 0.45% pt-ys. Non-users of antithrombotic drugs had an incidence of 0.08% pt-ys.

In relation to TTR results, in our population of self-managed patients we obtained a modest 63.6 ± 13.4%. In our previous clinical trial published in 2005 [12], we observed a similar figure. In agreement with Nilsson et al [9]*,* we found a better TTR in men that in women. Again, our current TTR results call into serious question the usefulness of evaluating the safety of oral anticoagulation by using percentage of in-range INR tests or time within target range. The decrease in the risk of complications may be explained by the empowerment of the patient [18, 19] and responsibility for making clinical decisions by themselves. More trained patients, such as patients who enter into a PSM regime, are more likely to adhere to the therapy, and hence achieve better results from the treatment [20–22].

The purpose of our study is mainly descriptive, since we had no comparison group. However, we have collected the main clinical outcomes in many publications on different models of anticoagulant therapy, in real world conditions to provide better objective (indirect) comparison between models. The different models and settings considered are: PSM of VKA therapy, conventional management of VKA therapy, management of VKA therapy in highly specialized centers, and results from direct oral anticoagulants (dabigatran, rivaroxaban, apixaban and edoxaban) in everyday clinical practice. Data are summarized in Tables 3, 4 and 5. We offer main results from different models of anticoagulant therapy for the three more frequent indications: atrial fibrillation [8, 11, 23–43], recurrent venous thromboembolism [8, 10, 23, 24, 44–48] and mechanical heart valves [8, 23, 24, 49–51].

**Table 3:** Incidence of major complications in different models of anticoagulant therapy in real world in patients with atrial fibrillation (AF)

							Incidence of major complications (% pt-ys)			
Author	Year	Drug	N	Mean age (years)	Total follow-up (pt-ys)	Mean follow-up (years)	Haemorrhagic	Thrombotic	Total^**c**^	Mortality
**PSM of VKA**										
Nagler [8] ^c^	2014	VKA	198	64.8	851^a^	4.3^a^	1.1	0.1	1.2^a^	2.5
Grove [11]	2018	VKA	534	63.1	1337	2.5	2.3	0.5	2.8	1.1
***Our study***	2018	VKA	120	65.3	726	6.0	1.6	0.4	2.0	2.3
**Conventional management of VKA in highly specialized centres**										
Wieloch [23]	2011	VKA	2491	74	2043	0.8^a^	2.6	1.4	4.0	NR
Sjöegren [24]	2015	VKA	51,229	72.1	143637^a^	2.8^a^	2.2	1.5	3.7	NR
Björck [25]	2016	VKA	40,449	72.5	65,424	1.6^a^	2.2	1.7	3.9	2.2
Esteve-Pastor [26]	2018	VKA	1361	76	8844^a^	NR	2.8	1.5	4.3^a^	6.2
**Conventional management of VKA**										
Sorensen [27]	2013	VKA	49,640	73.5	14892^a^	0.3^a^	4.3	0.7	5.0^a^	NR
Lauffenburger [28]	2015	VKA	43,865	71.4	43865^a^	1.0	5.2^a^	3.6^a^	8.8^a^	NR
Graham [29]	2015	VKA	67,207	100% > 65	19,382	0.3^a^	4.4	1.4	5.8^a^	3.8
Seeger [30]	2015	VKA	19,189	68.3	6448	0.3	6.2	1.3	7.4^a^	NR
Larsen [31]	2016	VKA	35,436	72.4	67328^a^	1.9	3.5	2.4	5.9^a^	7.2
Carmo [32]^b^	2016	VKA	501,019	NR	NR	NR	5.6	2.8	8.4^a^	6.1
Nielsen [33]	2017	VKA	38,893	71.0	89453^a^	2.3	3.1	2.7	5.8^a^	8.7
Chan [34]	2016	VKA	5251	71	4726^a^	0.9^a^	4.3	5.6	9.9	7.1
Li [35]	2017	VKA	38,470	70.9	NR	NR	7.5	3.1	10.6^a^	NR
Mentias [36]	2018	VKA	101,705	78.4	NR	NR	5.3	2.1	7.4^a^	6.7
Lee [37]	2018	VKA	12,183	70.7	10,965	0.9	3.6	3.9	7.5^a^	6.6
Gupta [38]	2018	VKA	7607	76.6	5051^a^	NR	4.7	1.9	6.6^a^	NR
Vinogradova [39]	2018	VKA	53,921	74.8	73,939	NR	2.5	1.7	4.2^a^	4.5
Chan [40]	2018	VKA	19,375	71.0	28481^a^	1.47	3.0	3.3	6.3^a^	9.1
**DOACs**										
Graham [29]	2015	Dabigatran	67,207	100% > 65	18,205	0.3^a^	4.3	1.1	5.4^a^	3.3
Lauffenburger [28]	2015	Dabigatran	21,070	67.5	21070^a^	1.0	3.2	1.7	4.9^a^	NR
Seeger [30]	2015	Dabigatran	19,189	68.7	8059	0.4	4.4	1.0	5.4^a^	NR
Larsen [31]	2016	Dabigatran	12,701	67.6	24131^a^	1.9^a^	2.0	1.8	3.8^a^	2.4
Graham [41]	2016	Dabigatran	52,240	100% > 65	15,524	0.3^a^	3.1	1.0	4.1^a^	2.2
Carmo [32]^b^	2016	Dabigatran	210,279	NR	NR	NR	3.9	1.6	5.5^a^	3.6
Chan [34]	2016	Dabigatran	5921	75	5329^a^	0.9^a^	2.6	3.7	6.3^a^	2.6
Nielsen [33]	2017	Dabigatran	8875	79.9	20,412	2.3	2.8	2.7	5.5^a^	9.1
Mentias [36]	2018	Dabigatran	21,979	75.8	NR	NR	3.4	1.5	4.9^a^	2.6
Gupta [38]	2018	Dabigatran	4129	73.0	3061^a^	NR	3.6	1.0	4.6^a^	NR
Vinogradova [39]	2018	Dabigatran	4534	74.7	5083	NR	2.2	1.7	3.9^a^	4.3
Chan [40]	2018	Dabigatran	20,079	75.0	31122^a^	1.55	2.0	2.7	4.7^a^	5.0
Laliberté [42]	2014	Rivaroxaban	3654	73.3	831^a^	0.23	3.3	4.6	7.9^a^	NR
Camm [43]	2016	Rivaroxaban	6784	71.5	6196^a^	0.9	2.1	0.8	2.9^a^	1.9
Larsen [31]	2016	Rivaroxaban	7192	71.8	13664^a^	1.9^a^	3.6	2.3	5.9^a^	6.7
Graham [41]	2016	Rivaroxaban	66,651	100% > 65	20,199	0.3^a^	4.5	0.8	5.3^a^	2.5
Chan [34]	2016	Rivaroxaban	3916	76	3524^a^	0.9^a^	3.4	3.1	6.5^a^	3.3
Nielsen [33]	2017	Rivaroxaban	3476	77.9	7994^a^	2.3	4.0	2.7	6.7^a^	13.5
Mentias [36]	2018	Rivaroxaban	23,177	75.7	NR	NR	4.7	1.4	6.1^a^	3.1
Gupta [38]	2018	Rivaroxaban	11,284	75.3	8303^a^	NR	4.4	1.3	5.7^a^	NR
Vinogradova [39]	2018	Rivaroxaban	13,597	75.8	12,679	NR	2.6	1.6	4.2^a^	5.5
Chan [40]	2018	Rivaroxaban	27,777	75.0	34443^a^	1.24	2.1	2.8	4.9^a^	6.0
Larsen [31]	2016	Apixaban	6349	71.3	5714	0.9	2.7	4.1	6.8	4.8
Nielsen [33]	2017	Apixaban	4400	83.9	4400^a^	1.0	3.9	4.0	7.9^a^	14.8
Li [35]	2017	Apixaban	38,470	70.9	NR	NR	4.5	2.1	6.6^a^	NR
Gupta [38]	2018	Apixaban	11,284	75.3	7000^a^	NR	2.9	1.0	3.9^a^	NR
Vinogradova [39]	2018	Apixaban	9199	76.5	7511	NR	1.5	1.8	3.3^a^	5.3
Chan [40]	2018	Apixaban	5843	76.0	4440^a^	0.76	1.5	2.3	3.8^a^	7.2
Lee [37]	2018	Edoxaban	4061	70.3	1218	0.3	2.3	3.2	5.5^a^	5.6
Grove [11]	2018	All	2671	63.6	5075	1.9^a^	2.3	1.6	3.9^a^	1.4

^a^ Approximated value

^b^ Meta-analysis; *NR* not reported

^c^ Total major complications were approximated as the sum of haemorrhagic plus thromboembolic, if not explicitly given in the publication

**Table 4:** Incidence of major complications in different models of anticoagulant therapy in real world in patients with recurrent venous thromboembolism (VTE)

							Incidence of major complications (% pt-yr)			
Author	Year	Drug	N	Mean age (years)	Total follow-up (pt-ys)	Mean follow-up (years)	Haemorrhagic	Thrombotic	Total^**b**^	Mortality
**PSM of VKA**										
Nagler [8]^b^	2014	VKA	451	48.5	1939^a^	4.3^a^	0.9	0.5	1.4^a^	1.1
Larsen [10]	2016	VKA	444	46.6	1953	6.9	0.4	1.3	1.7^a^	0.5
***Our study***	2018	VKA	213	47.5	1288	6.0	0.2	0.6	0.9	0.4
**Conventional management of VKA in highly specialized centres**										
Wieloch [23]	2011	VKA	1146	66	802	0.7^a^	2.6	1.8	4.4^a^	NR
Sjöegren [24]	2015	VKA	17,219	65.3	48213^a^	2.8^a^	2.0	1.1	3.1^a^	NR
Larsen [10]	2016	VKA	2220	46.8	9569	6.7	0.4	2.2	2.6^a^	1.1
**Conventional management of VKA**										
Ageno [44]	2016	VKA	2149	66.0	1719^a^	0.8^a^	2.3	2.6	4.9^a^	4.1
Sindet-Pedersen [45]	2017	VKA	6907	66	3453^a^	0.5	2.1	3.1	5.2^a^	NR
Coleman [46]	2017	VKA	32,244	43% > 60	16122^a^	0.5	1.0	3.5	4.5^a^	NR
Weycker [47]	2018	VKA	17,878	60	7437^a^	0.4	5.5	7.0	12.5^a^	NR
**DOACs**										
Ageno [44]	2016	Rivaroxaban	2619	59.0	1833^a^	0.7^a^	0.7	1.4	2.1^a^	0.5
Sindet-Pedersen [45]	2017	Rivaroxaban	5411	66	2705^a^	0.5	2.3	3.0	5.3^a^	NR
Coleman [46]	2017	Rivaroxaban	13,609	42% > 60	6804^a^	0.5	0.8	2.8	3.6^a^	NR
Berger [48]	2018	Rivaroxaban	3763	57.2	1656^a^	0.44	1.1	1.4	2.5^a^	NR
Weycker [47]	2018	Apixaban	17,878	60	7008^a^	0.4	4.2	5.8	10.0^a^	NR

The incidences in Ageno et al [44] and Sindet-Pedersen et al [45] were in % patients, not in % pt-ys

^a^ Approximated value; *NR* not reported

^b^ Total major complications were approximated as the sum of haemorrhagic plus thromboembolic, if not explicitly given in the publication

**Table 5:** Incidence of major complications in different models of anticoagulant therapy in real world in patients with mechanical heart valves

							Incidence of major complications (% pt-ys)			
Author	Year	Drug	N	Mean age (years)	Total follow-up (pt-ys)	Mean follow-up (years)	Haemorrhagic	Thrombotic	Total^**b**^	Mortality
**PSM of VKA**										
Mair [49]	2012	VKA	160	61.1	1376	8.6	2.2	0.6	2.8^a^	0.3^a^
Nagler [8]^b^	2014	VKA	356	55.0	1530^a^	4.3	0.4	1.5	1.9^a^	1.0
Christensen [50]	2016	VKA	615	57.2	2685	7.1	1.1	1.6	2.7^a^	1.1
***Our study***	2018	VKA	465	58.7	3236	7.0	1.3	1.1	2.4	1.8
**Conventional management of VKA in highly specialized centres**										
Wieloch [23]	2011	VKA	597	67	519	0.9^a^	2.3	2.7	5.0^a^	NR
Sjöegren [24]	2015	VKA	6997	66.1	19592^a^	2.8^a^	3.4	1.5	4.9^a^	NR
Christensen [50]	2016	VKA	3075	57.0	13,026	6.7	1.4	2.0	3.4	2.5
Grzymala [51]	2017	VKA	3831	63.3	18,022	4.7^a^	3.1	1.4	4.5	2.4
**Conventional management of VKA**										
Mair [49]	2012	VKA	260	66.3	2236	8.6	2.7	1.7	4.4^a^	1.9^a^

Data from Wieloch et al [23] and Sjöegren et al [24] relates to patients suffering from “heart valve disease”. We assume that the majority of them must be mechanical valves

^a^ Approximated value; *NR* not reported

^b^ Total major complications were approximated as the sum of haemorrhagic plus thromboembolic, if not explicitly given in the publication

In general, incidence of major bleeding, thromboembolic events, total severe complications and mortality are at least equal and probably better in the reports of PSM than in other models, including direct oral anticoagulants (DOACs) in those indications currently accepted (AF and VTE). Again, we must have caution in making conclusions, since the size of the studies, and the characteristics of the population included may not be always comparable. However, we want to pay attention in the excellent results reported for PSM in different countries and studies. To our knowledge there is only a unique study formally comparing outcomes of PSM of AVK against DOACs in patients suffering from atrial fibrillation. This is a very recent publication on a nationwide Danish study by means of a propensity matching method [11]. PSM of AVK patients had lower risk of all-cause and ischemic stroke compared to patients under DOAC treatment, whereas no significant differences were seen for major bleeding and mortality. In the current exponential growth of DOACs’ indication for AF worldwide, the results of Grove et al [11] along the reported by Nagler et al [8] and ours, point out the necessity of designing and conducting randomized controlled trials comparing PSM of AVK with DOACs.

### Strengths and limitations

Strength of our investigation are the long follow-up and the comprehensive and individualized search of available sources of clinical data, avoiding underestimating complications related to anticoagulation (including personal interview with 79% of the patients). All patients received the same training model and technology (POC).

Our study has several limitations. First of all, it is a single-center observational study with a retrospective analysis. Despite the exhaustive search, data on 8% of patients was lost and was not available for analysis. We may have lost information of patients who received assistance in private health centers and others that moved out from Catalonia. Also, patients who initiated PSM before 2008 (time when all clinical information was digitalized in our country) may have less available information.

There were no specific criteria of selection for patients to participate in the PSM programme. Most of included patients had been under conventional control for a long time and, therefore, had more experience with VKA treatment. Moreover, these patients received additional training at the start of PSM, thus this could be a reason of their better outcomes.

## Conclusions

We present clinical outcomes regarding incidence of major complications in a cohort of patients who self-managed their oral anticoagulant treatment with VKA. This is a one-center cohort representing 6019 pt-ys in total which includes different indications for VKA, mainly mechanical heart valves (50% of patients). In terms of clinically relevant outcomes, such as the global incidence of major complications and mortality by any cause, PSM in real life setting seems to be a very good alternative in properly trained patients.

## Data Availability

The datasets used and/or analysed during the current study are available from the corresponding author on reasonable request.

## Abbreviations

PSM: Patient self-management
VKA: Vitamin K antagonists
MHV: Mechanical heart valve
VTE: Recurrent venous thromboembolism
AF: Atrial fibrillation
TTR: Time in therapeutic range
OAT: Oral anticoagulant therapy
INR: International normalized ratio
TE: Thromboembolic events
TIA: Transitory ischemic attack
DOACs: Direct oral anticoagulants
